# Supplementary material for: Halide perovskite memristors as flexible and reconfigurable physical unclonable functions
Source: Nat Commun. 2021 Jun 17;12:3681. doi: 10.1038/s41467-021-24057-0 (PMC8211866; doi:10.1038/s41467-021-24057-0)
Supplement: Supplementary file 1 — Supplementary Information [file 41467_2021_24057_MOESM1_ESM.pdf]

Halide Perovskite Memristors as Flexible and Reconfigurable Physical Unclonable Functions

John et al.

Supplementary Note 1. Material Characterization

Possible sources of entropy in halide perovskite memristor PUFs (HP memPUFs):

HPs are ionic solids that exhibit relatively low activation energies for migration of both anions and cations due to the presence of vacancies, interstitials and antisite defects[1]. There are several mechanisms capable of modulating the conductivity and hence, memristive characteristics in HPs[2]. Unique to halide perovskites (HPs), is the co-existence and coupling of ionic and electronic components of current and capacitance, resulting in demonstrations of switchable majority carrier concentrations, giant dielectric constants, intrinsic localized doping and above bandgap photo-voltages[2–5]. Modulation via electrical and optical-fields gives rise to reversible local doping and phase segregation[6], resulting in tunable photoluminescence, and hysteretic resistive and capacitive behaviours. Like organic semiconductors, they allow facile deposition through solution processing while maintaining excellent semiconducting properties. Their unique orbital characteristics such as an anti-bonding valence band maximum (VBM) and a bonding conduction band minimum (CBM), result in defect tolerance and hence local doping and modulation of carriers[7]. The organic cation in the hybrid structure also allows for structural tunability, enabling modulation of the material's charge transport and band gap[8].

Electrochemical reaction of HPs with the metallic electrodes (e.g. Ag, Al) can result in the formation of metallic lead[9]/B-site cation[10] within the HP active layer or metal halides species (e.g. AgI) at the interface between HPs and the metal contact. This could lead to filament formation with modulatable conductivity depending on the type of metal and bias application[11]. Apart from that, the soft bonding and interconnected inorganic lattice also allows the ions to move relatively easily within the material's matrix. In particular, halide anions have been shown to exhibit much faster diffusion rate in comparison to the organic cation counterparts[1]. Such intrinsic halide vacancy migration can create tunable low resistance paths that can be broken on demand by applying voltages of opposite polarity, resulting in strong memresistance[12,13]. Lastly, the defect tolerance nature of these materials enables the motion of ions to subtly change the injection barriers at the perovskite-transport layer interfaces, hence modulating the electronic conductivity. Such ionic-electronic coupling effects have been shown to be capable of inducing interfacial doping near the electrodes. Specifically, field-assisted ionic drift and built-in voltage-assisted ionic back-diffusion can result in self p- and n-doping of the perovskite active layer and the transport layer interfaces upon application and removal of poling voltages, modulating the carrier injection barriers and hence the conductivity[3,11,14]. The dependence of defect concentrations on the fabrication methodology utilized (solvent choice, mixing time, fabrication conditions, annealing temperature, precursor source) as well as the morphology of the films formed, further provide a rich source of entropy for cryptographic primitives to generate device-specific secret bits.

Advantages of 1D HPs:

Here, we utilize a pyridinium-based HP material featuring molecular one-dimensional (1-D) lead-iodide lattices, namely PrPyr[PbI_3]. It was chosen over the 3D and 2D counterparts due to the higher electronic confinement imparted to the inorganic framework, which results in larger resistivity. This, in turn, is beneficial in obtaining superior performance of resistive switching[15]. Additionally, charge trap states present within the material can also be used to modulate the memristive characteristics. Such states are caused by point defects typically found in low-dimensional lead-halide hybrids as a result of the highly distorted nature of the constituent haloplumbate octahedra[16–18]. Furthermore, PrPyr[PbI_3]'s switching capability is also governed by the movement of iodide ion along the 1-D chain[19,20]. Such ionic migration is facilitated by the soft lead-iodide bonding as well as intrinsic iodide vacancy within the inorganic lattices. Following this halide movement, electrode polarization at the interface between the PrPyr[PbI_3] and interfacial layers is anticipated[21,22]. Although diffusion of the bulky organic species has lower probability, it is nonetheless expected to facilitate transient dipole formation upon application of bias as a result of the ease of polarizability of the pyridinium core[23–25]. We hypothesize that all of these factors would contribute to the stochasticity in conductive path formation in PrPyr[PbI_3] memristors as proxies to create PUFs that generate cryptographic keys with multiple challenge-response pairs.

The PrPyr[PbI3] films are deposited using a single-step solution-based method as detailed in the methods section. Physically, 1-D PrPyr[PbI_3] appears colourless and the UV-vis absorption spectrum of this material confirms its high bandgap nature (ca. 2.8 eV via extrapolation of the absorption edge; Supplementary Figure 1a, red curve). The feature is similar to those of other 1-D lead-iodide hybrids, in as much as the dielectric mismatch between organic and inorganic layers leads to strongly bound excitons, which leads to sharp excitonic absorption band at ca. 375 nm region[26,27]. Upon photoexcitation by 350 nm UV light, PrPyr[PbI_3] exhibits relatively strong photoluminescence (PL) at room temperature. The emission band is broad, with a maxima at 577 nm, an emission width of ca. 1.29 eV, and a full width at half maximum (FWHM) of ca. 540 meV (Supplementary Figure 1a, blue curve). Relative to its absorption onset, the observed PL exhibits a large Stokes shift of ca. 0.65 eV.

Such broad emission has been reported for related pyridinium-templated 1-D iodoplumbate systems and is attributed to a combination of band edge emissions and energy transfer from the excited state of the inorganic chains to the excited triplet state of the organic components[19,21,22]. However, it is also anticipated that coupling between excitons and phonons can occur (leading to self-trapped excitons) in hybrids having distorted inorganic lattices[28,29] (Supplementary Table 1). Time-resolved PL is additionally conducted (Supplementary Figure 1b) and it is found that the effective PL lifetime of PrPyr[PbI_3] is relatively short at room temperature (ca. 6.1 ns). This suggests that the broadband emission does not originate from triplet excited state(s) which could be obtained from excited-state structural reorganization of the 1-D

iodoplumbate chain moiety. In the material systems that involve such mechanism, long lifetime of hundreds to thousands of ns have been observed even at room temperature[30–32].

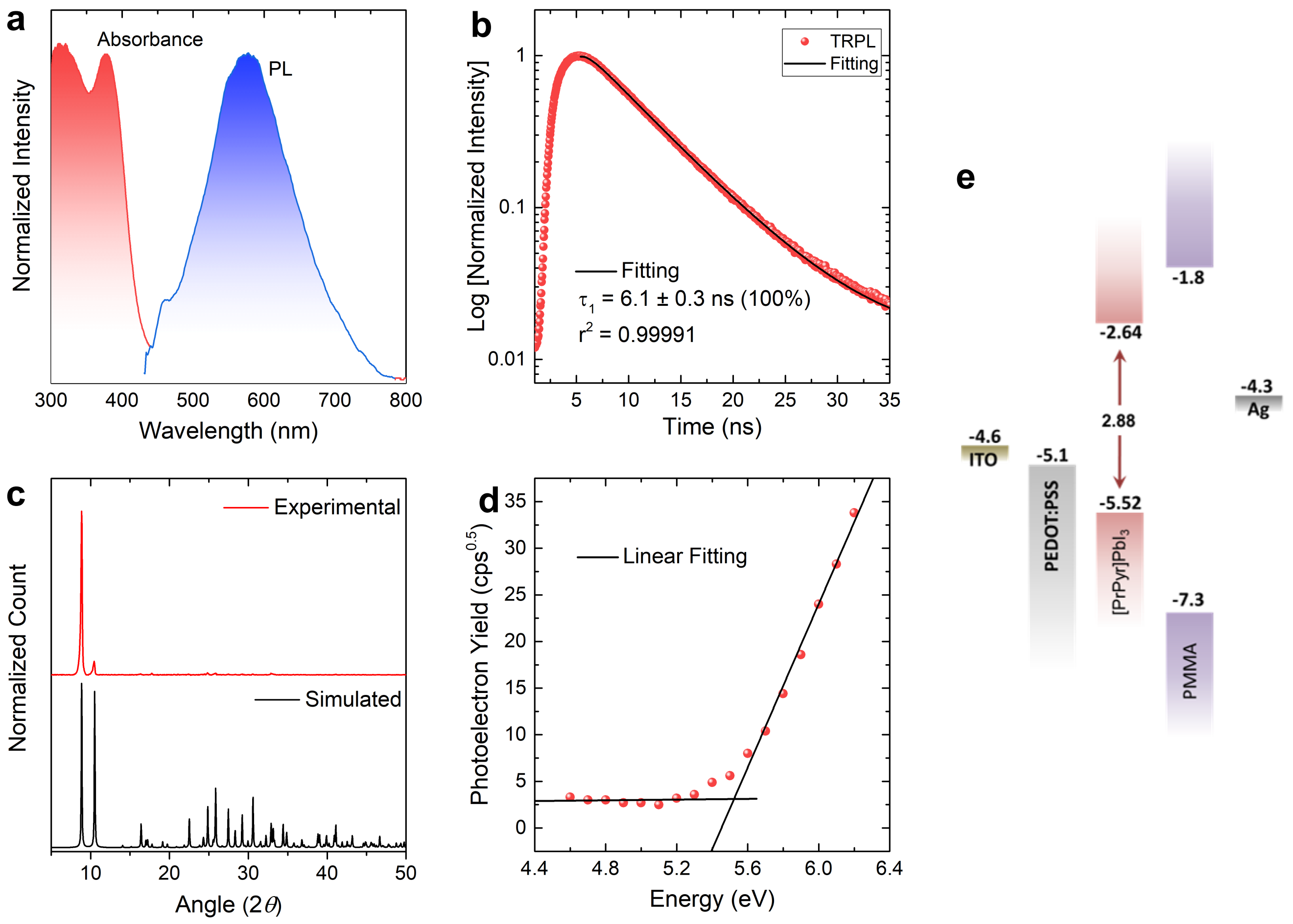

Supplementary Figure 1. Physical Characterization of PrPyr[PbI$_3$]. **a** UV-Vis absorption and photoluminescence (PL) spectra of 1-D PrPyr[PbI$_3$]. **b** Time-resolved photoluminescence (PL) spectrum of 1-D PrPyr[PbI$_3$], recorded at an emission wavelength of 575 nm. Monoexponential fittings afforded PL lifetimes of ca. 6.10 ns. **c** Glancing angle X-ray diffraction (GAXRD) pattern of 1-D PrPyr[PbI$_3$]. For comparison, the simulated room temperature (RT) powder XRD/PXRD pattern is included. **d** Photoelectron spectroscopy in air (PESA) measurements for PrPyr[PbI$_3$]. Solid lines represent linear fits of the two distinct domains and the intersection between them provides an estimate of the valence band energy of the 1-D material (i.e. ca. -5.52 eV). **e** Band energy diagrams of PrPyr[PbI$_3$], with the energies of the charge transport materials and electrodes comprising the memristor devices included for reference. Valence band energy is estimated using **c** photoelectron spectroscopy in air (PESA) measurement and the band gap is extracted from **a** UV-Vis absorbance spectrum.

Supplementary Table 1. Summary of distortion parameters for PrPyr[PbI3].

λ_{oct} $^{\alpha}$	$\sigma_{oct}^{2\beta}$	Δd (x10^{-4})$^{\gamma}$
1.0108	36.14	7.88

$^{\alpha}$Octahedral elongation (λ_{oct}); $^{\beta}$octahedral angle variance (σ_{oct}^{2}); and $^{\gamma}$octahedral bond length distortion (Δ_{oct}). The equations for each parameter and an explanation of their meanings are as the followings:

Octahedral elongation[33,34] $\lambda_{oct} = \frac{1}{6}\sum_{i=1}^{6}\left[\frac{d_i}{d_0}\right]^2$ …………………………………………….. (1)

Octahedral angle variance[33–36]: $\sigma^2{}_{oct} = \frac{1}{11}\sum_{i=1}^{12}(\alpha_i - 90)^2$ ……………………………… (2)

Bond length distortion[35]: $\Delta_{oct} = \frac{1}{6}\sum_{i=1}^{6}\left[\frac{d_i - d_m}{d_m}\right]^2$ ………………………………………… (3)

where d_i = Pb–Br bond length, d_m = average bond length, d_0 = center-to-vertex distance for a regular polyhedron of the same volume, and α_i = individual Br–Pb–Br angle. λ_{oct}, $\sigma^2{}_{oct}$ and Δ_{oct} provide a quantitative measure of polyhedral distortion, independent of the polyhedron effective size.

Glancing angle X-ray diffraction (GAXRD) measurement suggests that on top of substrates, PrPyr[PbI3] tends to crystallize with a preferred orientation. In particular, strong peaks at 2θ ca. 8.8 and 10.4° in Supplementary Figure 1c correspond to the orientations along (002) and (011) directions of the material's crystal lattices. Estimation of the valence band energies for PrPyr[PbI3] using photoelectron spectroscopy in air (PESA) yields a value of −5.52 eV (Supplementary Figure 1d), which is similar to the ones observed in other low-dimensional lead-halide hybrids[26,37]. Coupling this data with that obtained from the UV−vis measurement allows construction of the band energy diagrams shown in Supplementary Figure 1e, highlighting the effect of dimensionality quantization to the high bandgap nature of the material.

Supplementary Note 2. Cumulative Distribution Function (CDF)-Entropy Source Selection:

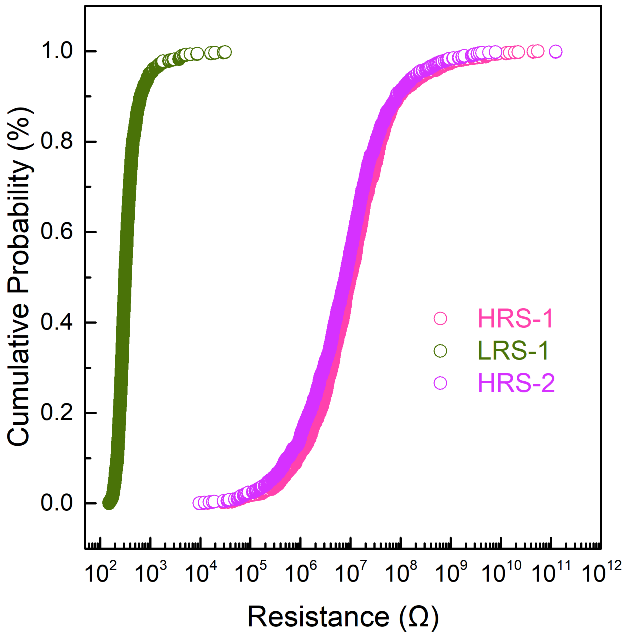

Supplementary Figure 2. Cumulative probability distribution plot of the LRS (green) and 2 HRSs (pink and violet) measured across 1024 devices respectively. Coefficient of variation is larger for HRS; hence we choose HRS as the entropy source.

Since conduction in the HRS is expected to be determined by the distribution of ions throughout the perovskite matrix, atomistic variations in the ionic distribution, tunnelling gap distance between the residual filament formation and the electrode, interfacial barriers, and charge trapping-detrapping dynamics can result in significant variations in the HRS between devices, serving as excellent sources of entropy. This is reflected in the cumulative distribution function (CDF) of the LRS and HRS. The coefficient of variation (CV), $\frac{\sigma}{\mu}$, expressing the spread of the distribution, is measured to be 2.54 for LRS and 9.07 for HRS. Larger variation in the HRS-1 distribution over LRS-1 justifies its selection as the entropy source[38].

Supplementary Note 3. Bit generation protocol and Write Back

Bit generation protocol

Our PUF consists of a 32×32 array of RRAM dot-point devices as shown in Fig. 3a, which we logically treat as a crossbar (future work will physically implement a crossbar). Consecutive 1-D HP RRAM pairs in one row are considered as a differential unit cell (2R cell). Hence, for an $A \times B$ array of RRAM, challenge bits can be used to select any of the A rows and any of the $B/2$ column pairs leading to maximum number of CRPs given by:

$$C_{max} = AB/2 \quad \ldots\ldots\ldots\ldots\ldots\ldots\ldots\ldots\ldots\ldots \text{(4)}$$

The selected row is connected to a bias voltage V_B while other rows are floating. The two selected columns are connected to a current comparator to produce a response bit b_R. This can be mathematically represented as:

$$\begin{aligned} b_R &= 1 \text{ if } V_B G^+(C) > V_B G^-(C) \\ &= 0 \text{ otherwise} \quad \ldots\ldots\ldots\ldots\ldots\ldots\ldots\ldots \text{(5)} \end{aligned}$$

where $G^+(C)/G^-(C)$ denotes the two differential conductances of the cell chosen by challenge C.

Differential current sensing is employed to generate a single bit. This differential design we adopt helps to cancel out first-order environmental dependencies. Since there are 1024 devices, we can obtain 512 independent pairs and therefore 512 bits. Independent pairs prevent exploitable correlation amongst pairs and increases the randomness of the 512-bit long bitstream. Row and column selections determine which pair of devices is selected in the 32×32 crossbar. The selected devices' currents are then fed into a comparator (such as sense amplifier) to generate one response bit. To further reduce the temporal fluctuations in resistance/read-out noise, the sensing margin is increased by adopting a write back process (explained below).

Write Back

Memristor cells with very closely spaced resistances are always vulnerable to noisy operating conditions that could result in bit flips and compromised crypto operations. To eliminate bit flips and the need for helper data, we exploit the widely separated bimodal resistance profiles of the HP memPUFs to reliably determine bits "1" and "0"[39]. Supplementary Figure 2 shows the resistance distribution of the HP memristor cells programmed into the 1st high resistance state (HRS-1) after the forming process. This distribution represents the entropy source for the HP memPUF. A complementary technique is used to increase the sensing gap. In a pair of memristor cells, the cell with higher current is reprogrammed/set to LRS while the other cell is left undisturbed in the HRS. The resultant large gap between these profiles due to the high on-off ratio of the HP memristor cells preserves the bit string across cycles and under varying operating conditions, allowing us to overcome bit-flip errors without fuzzy extractors or majority voting. This write back digitization strategy[39] does not affect the uniqueness in any way since the addresses of memristor cells

that need to be reprogrammed are based on the high analog entropy of HRS-1's distribution. Additionally, this approach also helps us overcome the issue of having memristor cells that are stuck at either the LRS or HRS, as they will be identified within one of the well-separated bimodal resistance profiles. This digitization also makes our HP memPUFs compatible with the CMOS digital peripheral circuits for facile generation of bitstreams from raw analog values.

We would also like to point out that the write-back may also be applied to each RRAM in a single ended fashion by using a median resistance to split the distribution[40]. This would result in twice the number of generated bits (C_{max} = AB). However, such implementations will be more susceptible to bit-errors due to changes in temperature, power supply voltage, etc., as well as require additional circuits to compute the median.

Supplementary Note 4. Reliability, Uniformity and Uniqueness on Raw Data

Some useful metrics for PUF are reliability (REL), uniformity (UF) and uniqueness (UQ)[41]. In short, UF is measured by the percentage of bit '1' or '0' in the response bit string with an ideal value of 50 %. UQ is measured by the inter-chip Hamming distance (inter-HD) defined as the HD between the responses of different PUF devices when subjected to the same challenge with an ideal inter-HD again as 50 %. Finally, REL (ideal value of 100 %) is quantified using the intra-chip HD (intra-HD) defined by the HD between responses measured on the same device at different times and conditions for the same challenge. A normalized value of intra-HD, defined as ratio of the number of error bits to the length of the response, is also called bit error rate (BER) which is (1 - REL). Practically, a large separation between the inter-HD and intra-HD reduces the false identification rates.

Uniformity (UF): UF is the number of times the PUF response is 1 out of m observations, as shown in the following equation:

$$\mathrm{UF} = \frac{1}{m}\sum_{i=1}^{m} \mathrm{r_i} \qquad (6)$$

where r_i is the ith response bit out of m observations. We ensure that uniformity is close to the 50 % mark (UF = 0.5) before we determine the reliability, in order to ensure there is no bias evident in the responses. Also, uniformity can also be thought of as the lower bound of machine learning (ML) prediction accuracy. In other words if uniformity were much further away from the ideal value of 50 %, there would not be any need to perform ML attacks and the attacker can plainly guess the response, whether 1 or 0, depending on the uniformity.

Supplementary Note 4 Supplementary Figure 3a shows a scatter plot of the average resistance difference of each differential pair ($\Delta R = R_{HRS,\,left} - R_{LRS,\,right}$) and the probability of outputting a "1" bit from that pair

in each trial across the switching cycles, for 1024 devices. A large value of $|\Delta R|$ indicates differential pairs that can produce reliable bits “1” or “0”, while a small value of $|\Delta R|$ suggests comparable resistance values that can produce either “0” or “1” due to read noise (resulting in bit flips). A mean UF of 49.02 % is obtained for the 512 bits as shown in Supplementary Figure 3b, which shows the generated bitstream is very close to being perfectly unbiased.

Uniqueness (UQ): When several instances of a single PUF design are fabricated, it is expected that the different instances will give different responses to the same challenge. The set of responses generated by each instance can be called the instance's signature. Uniqueness, UQ, of a PUF quantifies these signature differences and can be obtained using the following equation:

$$\mathrm{UQ} = \frac{2}{k(k-1)} \sum_{i=1}^{k-1} \sum_{j=i+1}^{k} \frac{\mathrm{HD}(\mathrm{r_i}, \mathrm{r_j})}{n} \quad \text{.......................... (7)}$$

where the term $\frac{2}{k(k-1)}$ represents the inverse of $\binom{k}{2}$ possible comparisons of the signatures amongst the different instances, and n is the length of the signature for each r_i and r_j comparison over which the HD is obtained. The ideal value of uniqueness is 50 % (UQ = 0.5) which suggests that different instances of a particular PUF have their own unique signature. If uniqueness were to be diverge away from this ideal value, it would mean the different instances of the PUF become less distinguishable from each other because the number of combinations $^{n}C_{r}$, where n is the response bit length and r is the number of bit difference between two signatures, will deviate from the maximum if $r \neq n/2$. As discussed in the main text, to calculate inter-HD or uniqueness for our 1024 devices, we create eight groups of 128 devices each. For each group we generate 64-bit keys out of 128 analog values, and we calculate the HD for $\binom{8}{2} = 28$ pairs of devices. We obtain a mean value of 48.3 % with 6.8 % standard deviation, showing excellent separation between inter and intra-HD.

Reliability (REL): Reliability is a measure of the consistency of a PUF's response regardless of environmental variations such as temperature, voltage, aging and temporal noise. The response at nominal environmental conditions, also called the golden response, is compared with the response at any other time/ voltage / temperature to determine if the responses are different (unreliable) or not (reliable). If r_0 is the golden response and r_e is the response at any condition other than the nominal environmental condition, then reliability REL of m observations can be mathematically expressed in terms of Bit Error Rate (BER):

$$\mathrm{REL} = 1 - \mathrm{BER} = 1 - \frac{1}{m}\left(\sum_{n=1}^{m} \mathrm{HD}(\mathrm{r_0}, \mathrm{r_{e,n}})\right) \quad \text{.......................... (8)}$$

We calculate the temporal, voltage and temperature-induced reliability, and the results are discussed in Supplementary Notes 5 and 6. The HD calculated in this case is also referred to as intra-HD since it is HD of responses within one chip. The 1 kb HP memPUF array bits are evaluated for 100 consecutive cycles to assess reliability (inversely BER) due to temporal noise. The resultant BER is 2.3 % without any correction schemes such as write-back, also known as native BER. Supplementary Note 6 shows further improvements attainable by different correction schemes.

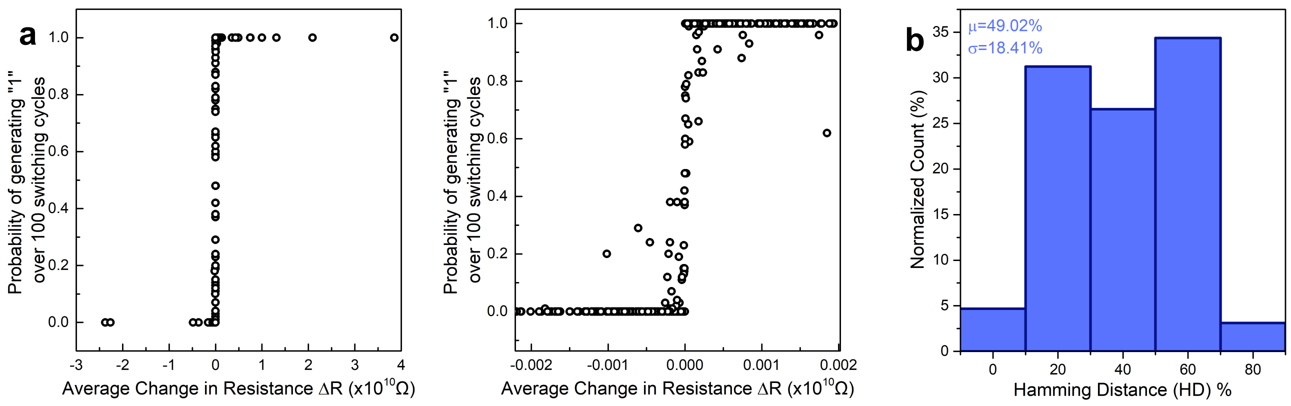

Supplementary Figure 3. a Scatter plot of differential resistance for 1024 devices. A centred plot demonstrates the equal likelihood of generating a 1 or 0 depending on positive or negative differential resistance. Note: The plot in the middle is a magnified view of the plot on the left. **b** Uniformity histogram for 64 groups of 8 bits each. A mean value of 49.02 % shows close to ideal uniformity and therefore excellent randomness.

Supplementary Note 5. Robustness to temporal, voltage and temperature fluctuations

The PUFs are analysed for robustness to temporal, voltage and temperature fluctuations as explained below. Robustness to temporal variations or transient effects are first evaluated. For assessing the robustness to temporal fluctuations, the testing procedure involves measuring the currents of all the devices for 100 cycles each. Temporal reliability is critical to be analysed from a PUF perspective since this determines the stability of the generated CRPs with time[42]. Since memristors are vulnerable to read variations over time, this analysis becomes even more critical. In this work, after we generate the CRP space in the enrolment phase, we experimentally measure the temporal stability of each of the 1024 devices of the HP memPUF array over 100 read cycles. The BER or intra-HD values presented in Supplementary Figure 5a shows the Bit-error rates (BER) due to temporal noise only.

For assessing the robustness to noisy voltage channels, the testing procedure involves measuring the currents of 16 representative devices with a read voltage of 0.1 V, and then measuring the current again with a read voltage of 0.15 V (Supplementary Figures 4a and 5b). We choose 0.15 V because it represents a 50 % deviation from the nominal value of 0.1 V. The measurements are repeated 100 times (hence, these results naturally captures the effects of both voltage and temporal fluctuations). This is in line with the standard test protocols adopted in literature[42]. We use a nominal read voltage of 0.1 V as a reference, and calculate the raw BER, without write-back, with respect to 0.1 V. Supplementary Figures 4ai-iii shows one example of the obtained bit map at 0.1 V and 0.15 V respectively. Without write-back, 3 of the bits are observed to flip (Supplementary Figure 4a-ii), but with write-back the bit flips are reduced to 0 (Supplementary Figure 4a-iii). Without write-back, we obtain an average BER of 19.25 % with the individual bit error rates plotted in Supplementary Figure 4a-iv. This value of BER is too large for the PUF to be used effectively in real-world applications. Fortunately, with write-back, the average BER with voltage and temporal fluctuations is reduced to 0 % (Supplementary Figure 4a-v).

To evaluate our HP memPUF array against temperature variations, the devices are heated up to 85°C and the data is collected at this constant thermal stress. The experimental measurements at room temperature are compared to the measurements under the 60°C and 85°C stress and the reliability of the devices are analysed without and with the write back step (Supplementary Figures 4b, c and 5c, d). At each temperature, the measurements are repeated 100 times and hence, these results naturally capture the effects of both temperature and temporal fluctuations. A checker board pattern of 16 representative devices is presented in Supplementary Figures 4b, c-i-v, along with the associated bit error rate (BER) values. The HP memPUFs are observed to be vulnerable to thermal stress and the extracted BER values are as high as 63.71 % without write back. However, once again, the write back strategy we adopt, allows us to maintain the integrity of the generated bits and CRP space at temperatures as high as 85°C by exploiting the widely separated bimodal resistance profiles of the HP memristors to reliably set the devices to their complimentary states.

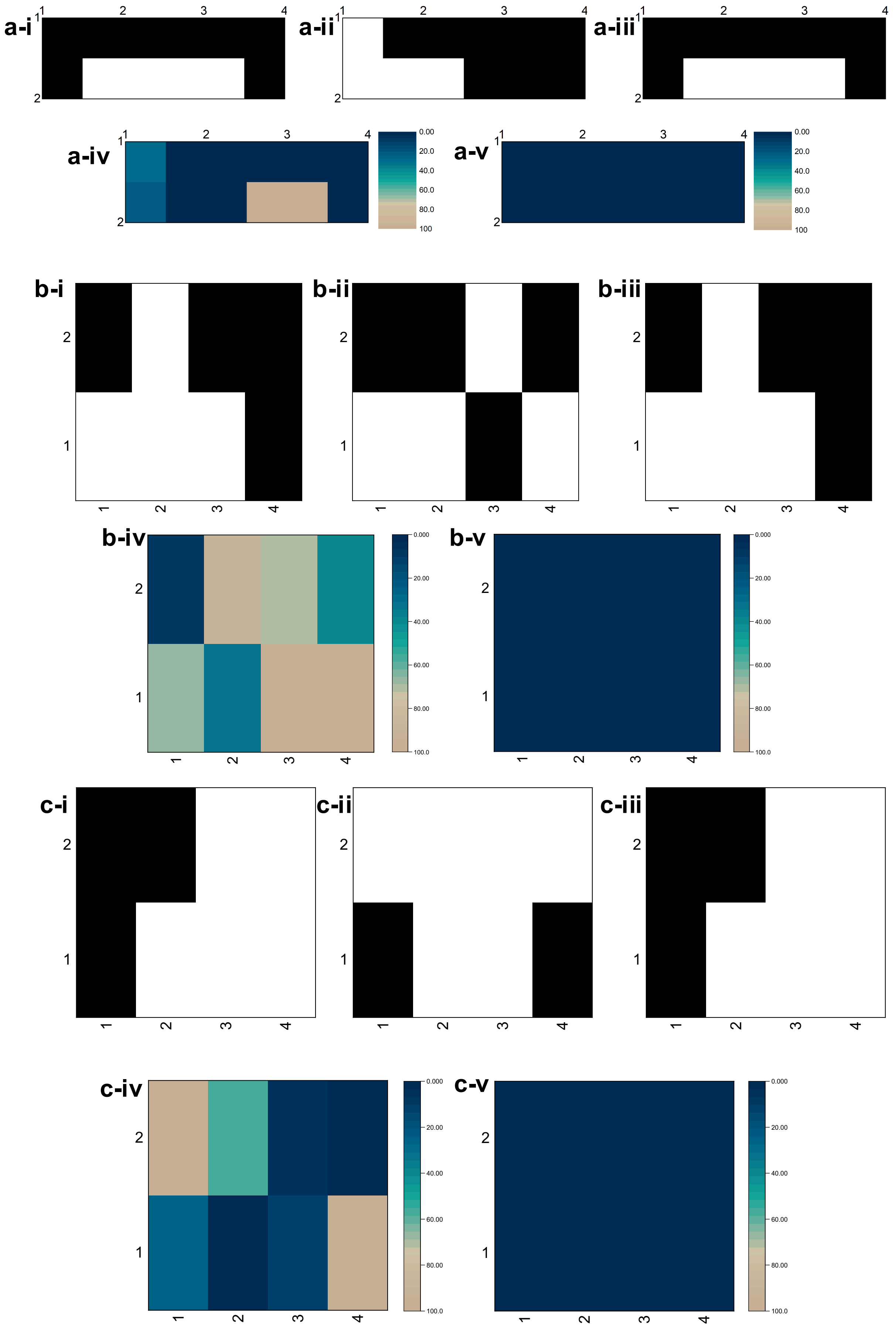

Supplementary Figure 4: **Robustness to temporal, voltage and temperature fluctuations. a** Reliability results with voltage and temporal fluctuations for 16 memristors. **a-i** shows the initial checkboard pattern obtained from 16 devices. Out of 100 measurements taken over time, the checkerboard patterns in **a-i**, **a-ii**,

a-iii are shown for one representative measurement. After applying a 50 % change in read voltage, checkerboard pattern in **a-ii** shows 3 bits flipping without write-back compared with **a-i**, and no bits flipping with write-back when **a-iii** is compared with **a-i**. BER color map shows errors across temporal and voltage variations in **a-iv** without write-back and no errors in **a-v** with write-back. 100 consecutive reads are done for both **a-iv** and **a-v**. **b-c** Reliability results with temperature and temporal fluctuations. **b-i** and **c-i** shows the initial checkboard pattern obtained from 16 devices. Out of 100 measurements taken over time, the checkerboard patterns in **b and c: i-iii** are shown for one representative measurement. After applying a thermal stress of **b** 60°C (**c** 85°C), the checkerboard pattern in **b-ii (c-ii)** shows 4 (3) bits flipping without write-back compared with **b-i (c-i)**, and no bits flipping with write-back when **b-iii (c-iii)** is compared with **b-i (c-i)**. BER color map shows errors across temporal and temperature variations in **b-iv** and **c-iv** without write-back and no errors in **b-v** and **c-v** with write-back. Note: 100 consecutive reads are done for **b-iv, b-v, c-iv and c-v**. The PUF instances shown in **b** and **c** are representative distinct responses.

Supplementary Note 6. BER Results and Strategies to improve BER

Since PUFs suffer from reliability issues due to environmental variations, we have to implement strategies in order to reduce the BER or intra-HD. Temporal Majority Voting (TMV) is a popular technique that reduces BER by applying majority voting across a chosen odd number of cycles and generating a single bit. Increasing the number of cycles improves the error reduction because it is akin to averaging over a greater number of samples to give a smoother result. However, TMV methods suffer from a strong trade-off between error reduction and latency incurred leading to generally modest improvements in BER only. Another technique we can use for RRAM PUFs is write-back as described in detail in Supplementary Note 3. Since write-back does not incur this latency-improvement trade-off, it is chosen for its superior performance.

Supplementary Figure 5 illustrates the BER results of the HP memPUFs under different strategies- (i) raw values without TMV, (ii) with different TMV settings and (iii) Write Back (WB). We measured data for the 1 kb array over 100 cycles, giving us 100 bits over temporal variation for each of the 1024 devices. For example with TMV-5, we generate a single bit by applying majority voting to every 5 successive bit groupings, resulting in 20 bits. We then measure BER for these 20 bits, which will be lower due to the averaging effect compared with 100 bits. Our results indicate that TMV is at most effective in reducing the native BER by 90-94 %. However, TMV is still not as effective as the write-back technique which can reduce BER to very near 0 %.

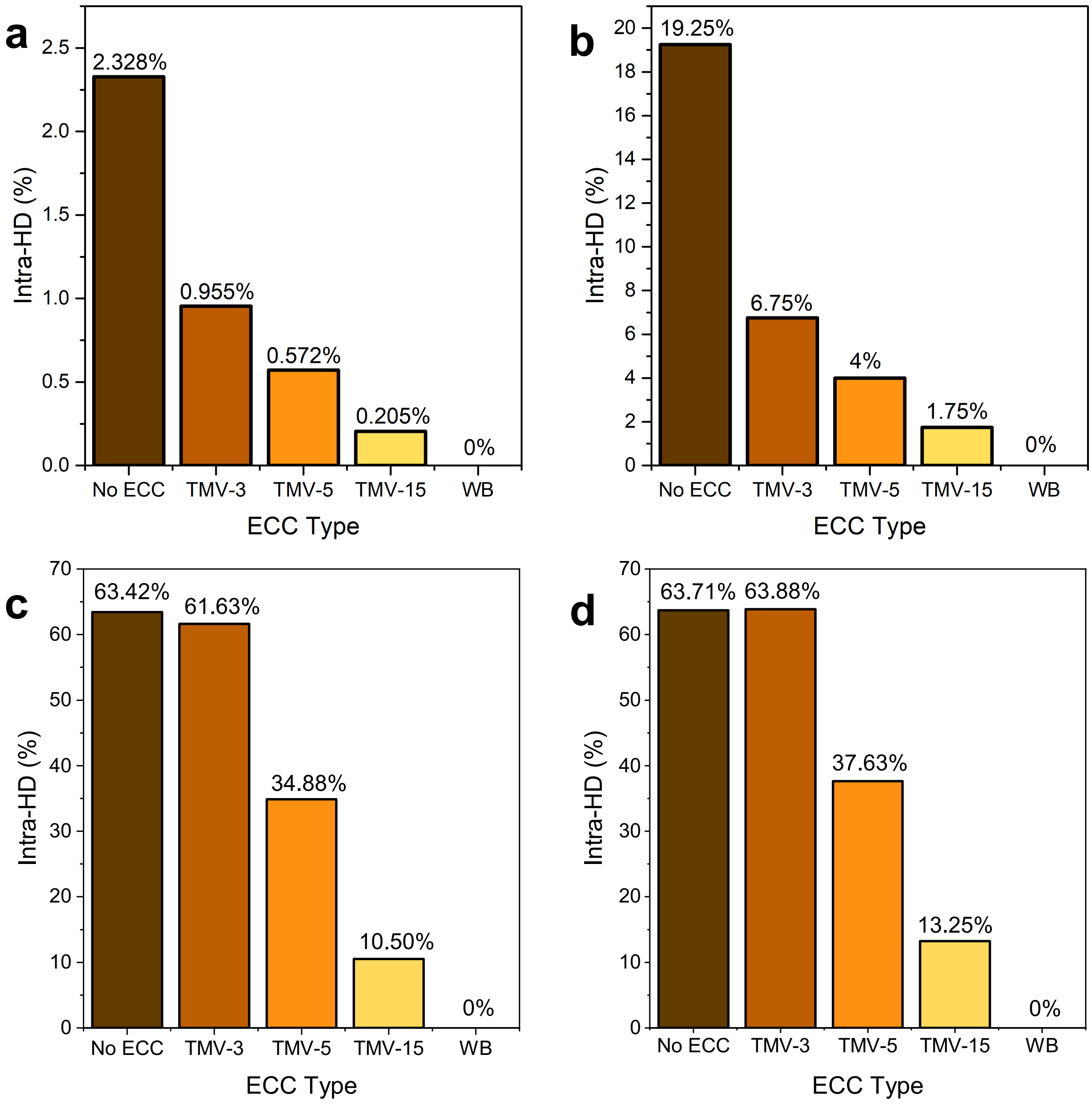

Supplementary Figure 5. Bit-error rates (BER) due to **a** temporal noise obtained by performing 100 consecutive reads, **b** 50 % change in read voltage, and temperature fluctuations of **c** 60°C and **d** 85°C. Improvements in BER by using Temporal Majority Voting and Write-Back (WB) are shown for all cases. WB reduces BER to 0 % and outperforms other TMV schemes.

Supplementary Note 7. Reconfigurability

Reconfiguration is possible for both strong and weak PUFs. For strong PUFs, authentication requires a secret challenge–response table to be established in the enrolment phase. This requires the server to securely communicate with the PUF device prior to any authentication rounds in a ‘‘secure bootstrapping’’ phase. In the deployment phase, once a CRP is used, it must be discarded and never used again. Therefore, the server must either store enough CRPs so that it will not run out, store an associated ‘‘secret model’’ that emulates the PUF CRP behaviour, or the PUF owner must periodically ‘‘update’’ the table at the secure site by replacing the old CRPs with new CRPs. Due to the large number of PUF devices that a single sever has to support and due to the finite storage space available on a server, the latter methodology of updating is the most feasible option. However, this in turn means that the physical functions of the PUF must be "updatable" or "reconfigurable". While logic reconfiguration strategies exist[43], they require additional area and power overheads. Moreover, most of the common CMOS PUF implementations that rely solely on manufacturing process variations exhibit a static CRP space and do not support true reconfiguration of their physical functions or physical reconfigurability.

Memristor-based PUFs have a special characteristic of irreversible reconfigurability due to their cycle-to-cycle variations, where each cycle constitutes a SET-RESET sequence. The cycle-to-cycle variation makes the CRP relationship completely different compared with the relationship before reconfiguration. The reconfiguration being irreversible means an attacker who was able to predict the response based on collected CRPs before reconfiguration will not be able to predict the response after reconfiguration. For weak PUFs, reconfiguration can produce a key that is orthogonal to the previous key and ensure freshness of the keys. This can prevent chosen plaintext attacks and make previously collected plaintext and ciphertext pairs redundant. Here, we report a truly physical reconfigurable HP memPUF whose reconfiguration HD is measured to be 40.06 % over 10 cycles. The mean value is 40.06 % with a standard deviation of 4.61 %. Since 10 cycles result in 10 keys, we compare $\binom{10}{2}$ times to get 45 measurements and calculate the statistics using these 45 HD values.

Supplementary Note 8. Strong PUF Design

The construction of strong PUF (Fig. 5a) requires summing up of current values of cells in rows where row challenge bit is 1 and compare summed up currents in a column pair with each other to generate a response bit. For an $A \times B$ crossbar, the challenge would thus be both the row addresses (A bits) and column pair selection ($\log_2(B/2)$). In this manner an exponential number of CRPs can be generated. For this design we do not write-back and use the raw HRS or LRS conductance values, though write-back can be used as done for the weak PUF. A 1 kb array can be arranged into a square-shaped 32 × 32 crossbar such that $A= B= 32$. As a result the maximum number of CRPs (C_{max}) we can get are:

$$C_{max} = 2^{32} \times \frac{32}{2} = 6.87 \times 10^{10}$$

Although we have a maximum of $\binom{32}{2}$ ways we can choose column pairs, we only choose 16 column pairs because they are independent of each other. If dependant column pairs are chosen such as A-B, B-C and A-C, the attacker can glean the relationship of, for example, A-C if he/she knows the CRPs of the other two column pairs[44].

Recurrent construction to combat Machine learning (ML) attacks :

A further method of information leakage for strong PUF is through modelling attacks since a very large number of CRPs used for authentication purposes may be available to an attacker using man-in-the-middle attacks[45–47]. Machine learning (ML) attacks can then be mounted by using algorithms to model the correlations between CRPs. Due to the linear addition of conductances in our strong PUF, it is expected to be vulnerable to such attacks, similar to the problem faced by arbiter PUFs[46]. Using 128,000 CRPs for training and 28,000 CRPs for testing, a variety of ML algorithms were used to model the PUF response with bagged trees achieving the highest test accuracy of ~ 86 % (Fig. 5b).

One method of improving ML attack resistance at the expense of using more devices is to create multi-layer PUFs[48] by cascading crossbar PUFs to implement more obfuscation in the challenge-response relationship. Instead, to save area and get better resistance in the same number of devices, we chose to fold the multiple layer obfuscation into the same PUF crossbar using recurrence (Fig. 5c). Similar trade-offs in area vs speed is achieved in the design of algorithmic vs pipeline analog to digital converters as well[49]. The usage of recurrence in PUFs involves generating an intermediate response from the PUF which is then fed back and XOR'ed with the challenge to obtain the final response bit[44]. Testing ML attacks on these new CRPs with the same training-testing split produces a maximum attack accuracy of 52 % (Fig. 5d), far lower than the results without recurrence and close to the 50 % accuracy of random guessing.

Training with 10x increased number of samples, i.e. 10^6 samples, reveals similar trends of better attack resilience with recurrence. Firstly, it is noted that the trend of better attack resilience with recurrence still holds true with higher number of CRPs (Supplementary Figure 6 b vs a). Secondly, it can be seen from Supplementary Figure 6b that the rate of increase of ML attack accuracy drops sharply on increasing the number of samples and is expected to saturate at values < 60 %. Although Supplementary Figure 6b shows the accuracies increasing with number of CRPs for the Bagged Trees and Adaboost algorithms, the rate of increase is approximately 0.7 % for every doubling of training CRPs even after collecting around 1.6 million CRPs. For the worst case assuming constant slope and extrapolating from 2^{20} CRPs as shown in Supplementary Figure 6c, for which the accuracy is 54 %, the attacker would need to collect 2^{88} CRPs to increase the accuracy by 46 % to 100 %. With an accuracy of 100 %, the PUF has been successfully impersonated by the attacker because the accuracy has increased beyond the reliability of our PUF[50]. However, the rate at which CRPs are collected do not typically exceed 2Mbps[51]. Nevertheless, even if we assume an aggressive 10Mbps CRP collection rate, it will take 9.8×10^{11} years for the attacker to collect 2^{88} CRPs and achieve close to 100 % accuracy, demonstrating that our HP memPUFs are secure against the aforementioned ML attacks.

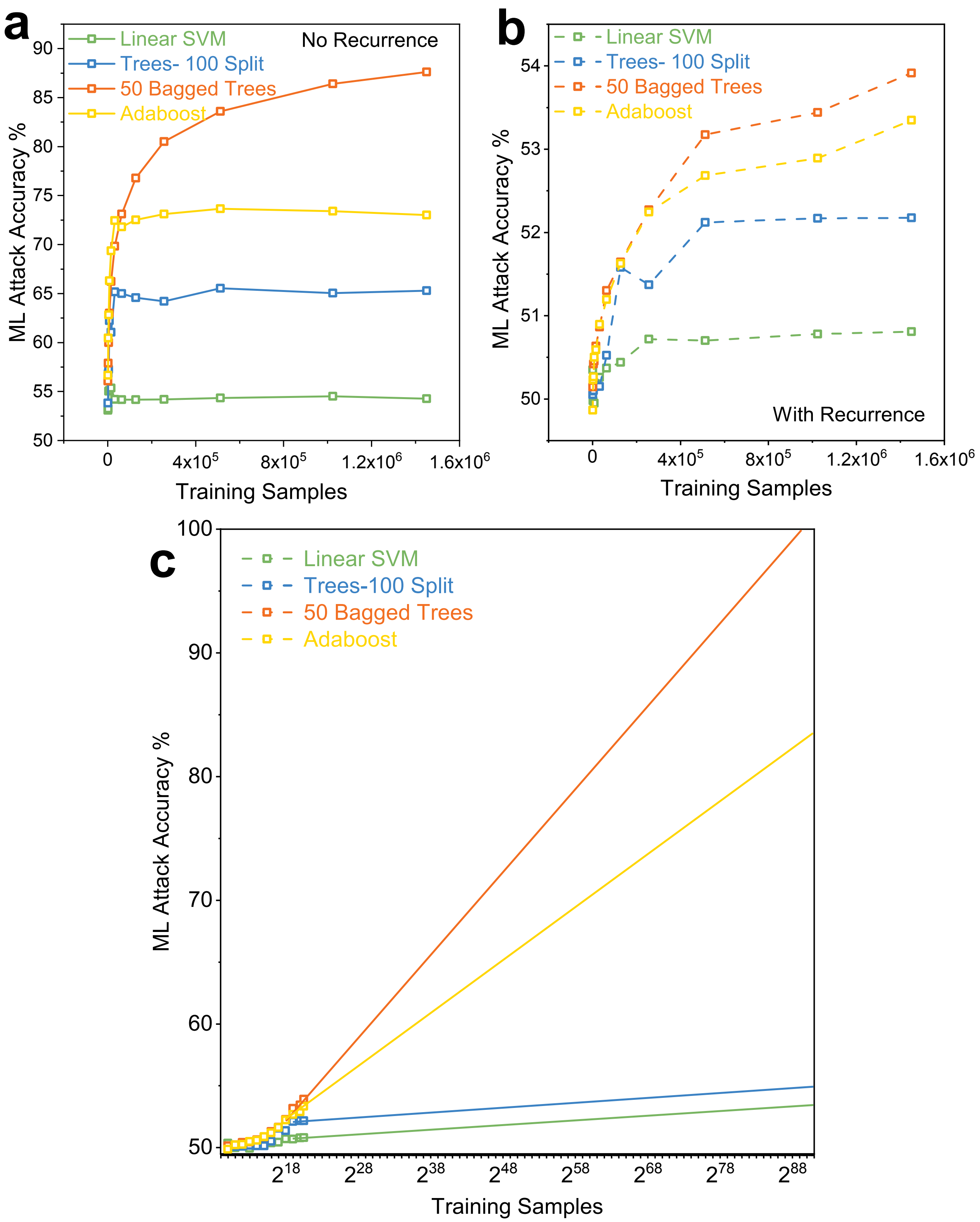

Supplementary Figure 6. 1-D HP Strong memPUF resilient to Machine Learning Attacks. a Machine learning (ML) results for strong PUF without recurrence upon training with 10^6 samples. Accuracies close to 90 % reveals the HP memPUF to be highly susceptible to such attacks. **b** ML results for strong PUF with recurrence upon training with 10^6 samples. Accuracies reduce to almost wild guess probability with recurrence compared to **a**, proving that recurrence improves resistance to ML attacks. **c** Extrapolation of the attack accuracy for very large number of training samples. Plot shows accuracy reaching almost 100 % for 2^{88} CRPs, however collecting these many CRPs in a reasonable amount of time is not feasible.

Supplementary Note 9. Power estimation

We estimate power consumption for all the three constructions: weak PUF, strong PUF without recurrence and strong PUF with recurrence by running SPICE simulations. For the weak PUF, we only select two devices after write-back and compare their currents. We assume the current in HRS is ~ 100 nA and the current in LRS ~ 1 μA[48]. We use a latch-based comparator with offset-cancellation[44] whose power consumption is 3.61 μW and propagation delay is 8.3 ns. As a result we obtain energy/bit of 29.9 fJ/bit. For the strong PUF without recurrence, we assume a randomly chosen challenge activates on average half the number of rows, i.e., 16 rows or 16 devices per column. Since we use two columns to generate a single bit, the simulated energy/bit obtained is 51.4 fJ/bit. The strong PUF with recurrence consumes more power due to the extra power burned during intermediate response generation and the recurrence loop. Since we generate two intermediate response bits and apply recurrence once, we are selecting column pairs three times and burning power in the comparator three times. Furthermore, the 32 XOR gates consume a minimal energy of 0.24 fJ as well. As a result, we obtain a value of 0.102 pJ for the energy/bit of the strong PUF with recurrence.

Supplementary Note 10. National Institute of Standards and Technology (NIST) Tests

We run NIST tests (Supplementary Table 2) to check the randomness properties of our HP memPUF. Since we only have 1024 bits from 1024 devices for use as a weak PUF, we apply post-processing to generate few million bits necessary to run these tests. MATLAB is used for the subsequent analysis and bit generation. The weak PUF bit generation algorithm exploiting combinations is used to generate approximately 12.5 million bits from the 1024 raw conductance values[52]. Each bit is generated by comparing a group of "N" summed up conductance values with another such group. In our case we choose "N" = 3 resulting in a total number of CRPs, $C_{max} = {}^{1024}C_6$. Theoretically, "N" can be any number however if "N" gets too large, the speed of summation calculation reduces drastically. The two groups to be compared against each other are chosen at random from the 1024 conductance values, making sure there are no duplicates. Once the bits are generated, they are passed into the NIST test suite and the results are generated.

We also run NIST tests to check the randomness quality of the strong PUF responses as reported in Table 1 of main text. The strong PUF response generation scheme is discussed in Supplementary Note 8. The results demonstrate that both the weak and strong HP memPUFs qualify as having random enough bitstreams.

Supplementary Table 2: Results of NIST test for weak PUF construction. Approximately 12.5 million bits are generated using a post-processing technique of comparing groups of 3 devices' summed up conductances.

Test	P-value	No. of runs	Pass No.	Pass?
Frequency	0.3838	200	198	Yes
Block Frequency	0.6059	200	199	Yes
Cumulative Sums	0.5141	200	198	Yes
Runs	0.9357	200	200	Yes
Longest Run	0.0179	200	195	Yes
FFT	0.6163	200	197	Yes
Serial	0.6267	200	200	Yes
Linear Complexity	0.1223	200	198	Yes
Rank	0.0805	100	99	Yes
Approximate Entropy	0.8832	50	50	Yes
Random Excursions	-	6	6	Yes
Random Excursions Variant	-	6	6	Yes
Non Overlapping Template	0.4559	50	49	Yes
Overlapping Template	0.5955	200	199	Yes
Universal	0.2133	20	20	Yes

Supplementary Note 11: Comparison with Literature

Supplementary Table 3. Benchmark comparison

Reference	Miller, Avi, et al. 2019 IEEE Custom Integrated Circuits Conference (CICC). IEEE, 2019.[53]	Pang, Yachuan, et al. IEEE Electron Device Letters 38.2 (2017): 168-171.[54]	Liu, Rui, et al. 2016 IEEE International Symposium on Hardware Oriented Security and Trust (HOST). IEEE, 2016.[55]	Pang, Yachuan, et al. *2019 IEEE International Solid-State Circuits Conference -(ISSCC)*. IEEE, 2019.[56]	Nili, H., et al. Nature Electronics 1.3 (2018): 197-202.[48]	Jiang, H., et al. Nature Electronics 1.10 (2018): 548-554.[57]	**This work**
Technology	SRAM	TaO_x /$HfAl_yO_x$ memristor	HfO_2 memristor	HfO_2 memristor	TiO_{2-x} memristor	HfO_2 memristor	**1-D Halide perovskite PrPyr[PbI_3] memristor**
Methodology	-	Variations in HRS	Variations in HRS	Variations in HRS	I-V non-linearity variation	Variations in LRS	**Variations in HRS**
Source of entropy	V_t mismatch	Oxygen Vacancies	Oxygen Vacancies	Oxygen Vacancies	Oxygen Vacancies	Oxygen Vacancies	**Ion migration, electrochemical metallization, interfacial doping**
Array Size	800b	1 kb	1 kb	8 kb	2x10x10	128 × 64	**1 kb**
Uniformity (%)	51.4	-	-	50.01 $\pm$ 2.87	49.5 - 50.0	-	**49.02**
Uniqueness/ Interclass HD (%)	50.27	49.8	~ 42	49.99 $\pm$ 4.35	50.0 $\pm$ 6.26	50.06 $\pm$ 4.52	**48.3 $\pm$ 6.8**
Intraclass HD (%)	19.7 % (pre-tilt), < 7.4e-10 (post-tilt)	1	0	0	1.22 $\pm$ 1	13.82 $\pm$ 6.57	**2.33 $\pm$ 8.08 (no WB); 0 $\pm$ 0 (with WB)**
Reconfigurability	No	No	No	Yes	Yes	Yes	**Yes**
NIST Tests	No	No	No	Yes	Passes 13 tests	No	**Passes 15 tests**
Flexible	No	No	No	No	No	No	**Yes**

This work- in bold

Supplementary Note 12: Future-proofing for large crossbar implementations

We expect all our findings to remain valid even with future implementations of large crossbar arrays of HP memristors. The only scenario where our experimental measurements on dot point devices could differ from crossbar implementations would be when the resistance of the contacts lines in the crossbar interferes with our measurements of the high (HRS) and low resistance states (LRS). To determine this, a small 8x8 crossbar array of HP memPUFs was fabricated and the contact resistance of the lines (thickness = 50 nm, width = 200 µm and length: 17.5 mm) were extracted. The resistance of the top and bottom electrodes were found to be equal to 65 ohms, far lower than our LRS of ~ 350-400 ohms (Supplementary Figure 7a). Further, dividing this by 8 results in an unit cell resistance of the wiring to be only ~ 8 ohms. Hence, we expect our results and approach to hold well with future implementations of large crossbar arrays of halide perovskite memristors. We also show below the representative IV characteristics of a dot point and crossbar HP memristor with the same device area (Supplementary Figures 7b-c). Both configurations yield similar results per se. However, we are currently limited by the poor yield of large crossbar arrays of halide perovskite memristors with reasonable endurance and hence, we implement dot point arrays for this work. With focussed engineering efforts to improve the yield of perovskite crossbar memristors, we expect to experimentally fabricate large-scale halide perovskite PUF chips in the near future.

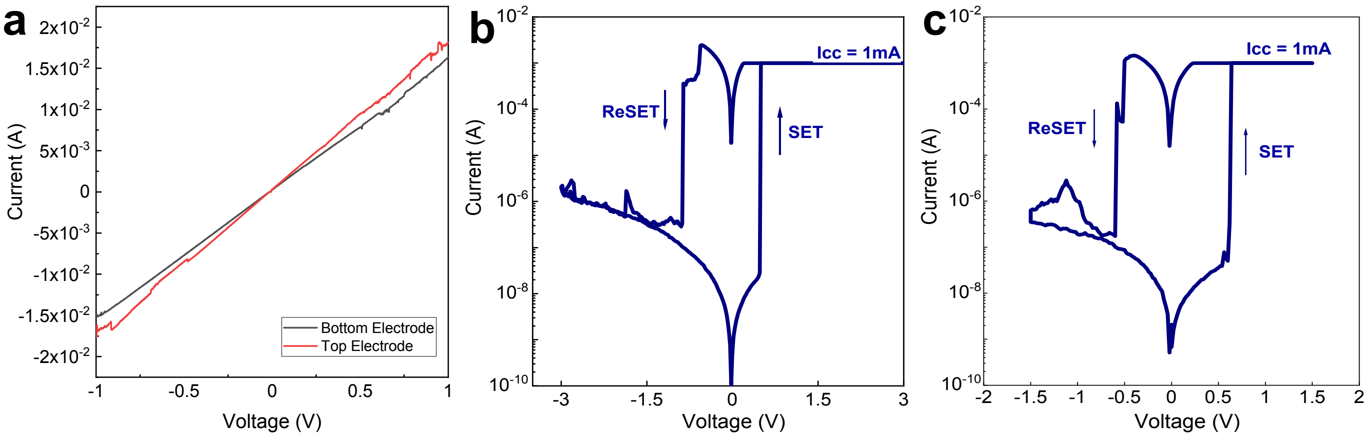

Supplementary Figure 7. a Contact line resistance of the top and bottom electrodes of a 8x8 crossbar array of HP memristors. Representative IV characteristics of a **b** dot point and **c** crossbar HP memristor with the same device area.

Supplementary References:

1. Azpiroz, J. M., Mosconi, E., Bisquert, J. & De Angelis, F. Defect migration in methylammonium lead iodide and its role in perovskite solar cell operation. *Energy Environ. Sci.* **8**, 2118–2127 (2015).
2. Harikesh, P. C., Febriansyah, B., John, R. A. & Mathews, N. Hybrid organic–inorganic halide perovskites for scaled-in neuromorphic devices. *MRS Bull.* **45**, 641–648 (2020).

3. John, R. A. *et al.* Ionotronic Halide Perovskite Drift-Diffusive Synapses for Low-Power Neuromorphic Computation. *Adv. Mater.* 1805454 (2018).
4. Xiao, Z. *et al.* Giant switchable photovoltaic effect in organometal trihalide perovskite devices. *Nat. Mater.* **14**, 193 (2015).
5. Harikesh, P. C. *et al.* Cubic NaSbS2 as an Ionic–Electronic Coupled Semiconductor for Switchable Photovoltaic and Neuromorphic Device Applications. *Adv. Mater.* 1906976 (2020).
6. Bischak, C. G. *et al.* Origin of reversible photoinduced phase separation in hybrid perovskites. *Nano Lett.* **17**, 1028–1033 (2017).
7. Harikesh, P. C. *et al.* Doping and Switchable Photovoltaic Effect in Lead-Free Perovskites Enabled by Metal Cation Transmutation. *Adv. Mater.* **30**, 1802080 (2018).
8. Amat, A. *et al.* Cation-induced band-gap tuning in organohalide perovskites: interplay of spin–orbit coupling and octahedra tilting. *Nano Lett.* **14**, 3608–3616 (2014).
9. Zhao, L. *et al.* Redox chemistry dominates the degradation and decomposition of metal halide perovskite optoelectronic devices. *ACS Energy Lett.* **1**, 595–602 (2016).
10. Yang, J.-M. *et al.* Perovskite-related (CH 3 NH 3) 3 Sb 2 Br 9 for forming-free memristor and low-energy-consuming neuromorphic computing. *Nanoscale* **11**, 6453–6461 (2019).
11. John, R. A. *et al.* Diffusive and Drift Halide Perovskite Memristive Barristors as Nociceptive and Synaptic Emulators for Neuromorphic Computing. *Adv. Mater.* 2007851 (2021).
12. Xu, W. *et al.* Organometal halide perovskite artificial synapses. *Adv. Mater.* **28**, 5916–5922 (2016).
13. Zhu, X. & Lu, W. D. Optogenetics-inspired tunable synaptic functions in memristors. *ACS Nano* **12**, 1242–1249 (2018).
14. Xiao, Z. & Huang, J. Energy-efficient hybrid perovskite memristors and synaptic devices. *Adv. Electron. Mater.* **2**, 1600100 (2016).
15. Kim, S. *et al.* Dimensionality dependent plasticity in halide perovskite artificial synapses for neuromorphic computing. *Adv. Electron. Mater.* **5**, 1900008 (2019).
16. Cortecchia, D. *et al.* Broadband emission in two-dimensional hybrid perovskites: The role of structural deformation. *J. Am. Chem. Soc.* **139**, 39–42 (2017).
17. Wu, G. *et al.* A one-dimensional organic lead chloride hybrid with excitation-dependent broadband emissions. *ACS Energy Lett.* **3**, 1443–1449 (2018).
18. Zhou, J. *et al.* Broad-Band Emission in a Zero-Dimensional Hybrid Organic [PbBr6] Trimer with Intrinsic Vacancies. *J. Phys. Chem. Lett.* **10**, 1337–1341 (2019).
19. Duan, H.-B., Yu, S.-S., Tong, Y.-B., Zhou, H. & Ren, X.-M. Two in one: switchable ion conductivity and white light emission integrated in an iodoplumbate-based twin chain hybrid crystal. *Dalt. Trans.* **45**, 4810–4818 (2016).
20. Eames, C. *et al.* Ionic transport in hybrid lead iodide perovskite solar cells. *Nat. Commun.* **6**, 1–8

(2015).

21. Duan, H.-B., Yu, S.-S., Liu, S.-X. & Zhang, H. An inorganic–organic hybrid crystal with a two-step dielectric response and thermochromic luminescence. *Dalt. Trans.* **46**, 2220–2227 (2017).
22. Duan, H.-B., Yu, S.-S., Liu, S.-X. & Zhang, H. A multi-functional iodoplumbate-based hybrid crystal: 1-propyl-4-aminopyridinium triiodoplumbate. *RSC Adv.* **7**, 23234–23237 (2017).
23. Chaban, V. V & Prezhdo, O. V. Polarization versus temperature in pyridinium ionic liquids. *J. Phys. Chem. B* **118**, 13940–13945 (2014).
24. Duan, X.-M. *et al.* Second-order hyperpolarizability of pyridinium cations. *Chem. Lett.* **26**, 247–248 (1997).
25. Wąsicki, J., Fojud, Z., Czarnecki, P. & Jurga, S. Polarisation and energy barriers in ferroelectric pyridinium perchlorate. *Ferroelectrics* **368**, 63–71 (2008).
26. Febriansyah, B. *et al.* Inducing panchromatic absorption and photoconductivity in polycrystalline molecular 1D lead-iodide perovskites through π-stacked viologens. *Chem. Mater.* **30**, 5827–5830 (2018).
27. Pradeesh, K., Agarwal, M., Rao, K. K. & Prakash, G. V. Synthesis, crystal structure and optical properties of quasi-one-dimensional lead (II) iodide: C14H18N2Pb2I6. *Solid state Sci.* **12**, 95–98 (2010).
28. Mao, L. *et al.* Structural diversity in white-light-emitting hybrid lead bromide perovskites. *J. Am. Chem. Soc.* **140**, 13078–13088 (2018).
29. Cui, B.-B. *et al.* Locally collective hydrogen bonding isolates lead octahedra for white emission improvement. *Nat. Commun.* **10**, 1–8 (2019).
30. Zhou, C. *et al.* Blue emitting single crystalline assembly of metal halide clusters. *J. Am. Chem. Soc.* **140**, 13181–13184 (2018).
31. Zhou, C. *et al.* Luminescent zero-dimensional organic metal halide hybrids with near-unity quantum efficiency. *Chem. Sci.* **9**, 586–593 (2018).
32. Zhou, C. *et al.* Facile preparation of light emitting organic metal halide crystals with near-unity quantum efficiency. *Chem. Mater.* **30**, 2374–2378 (2018).
33. Robinson, K., Gibbs, G. V & Ribbe, P. H. Quadratic elongation: a quantitative measure of distortion in coordination polyhedra. *Science* **172**, 567–570 (1971).
34. Thomas, N. W. Crystal structure–physical property relationships in perovskites. *Acta Crystallogr. Sect. B Struct. Sci.* **45**, 337–344 (1989).
35. Ertl, A. *et al.* Polyhedron distortions in tourmaline. *Can. Mineral.* **40**, 153–162 (2002).
36. Fleet, M. E. Distortion parameters for coordination polyhedra. *Mineral. Mag.* **40**, 531–533 (1976).
37. Febriansyah, B. *et al.* Improved Photovoltaic Efficiency and Amplified Photocurrent Generation in Mesoporous n= 1 Two-Dimensional Lead–Iodide Perovskite Solar Cells. *Chem. Mater.* **31**, 890–898 (2019).

38. Chen, A. Comprehensive assessment of RRAM-based PUF for hardware security applications. in *2015 IEEE International Electron Devices Meeting (IEDM)* 10–17 (IEEE, 2015).
39. Che, W., Plusquellic, J. & Bhunia, S. A non-volatile memory based physically unclonable function without helper data. in *2014 IEEE/ACM International Conference on Computer-Aided Design (ICCAD)* 148–153 (IEEE, 2014).
40. Liu, R., Wu, H., Pang, Y., Qian, H. & Yu, S. Experimental characterization of physical unclonable function based on 1 kb resistive random access memory arrays. *IEEE Electron Device Lett.* **36**, 1380–1383 (2015).
41. Maiti, A., Gunreddy, V. & Schaumont, P. A systematic method to evaluate and compare the performance of physical unclonable functions. in *Embedded systems design with FPGAs* 245–267 (Springer, 2013).
42. Gassend, B., Lim, D., Clarke, D., Van Dijk, M. & Devadas, S. Identification and authentication of integrated circuits. *Concurr. Comput. Pract. Exp.* **16**, 1077–1098 (2004).
43. Katzenbeisser, S. *et al.* Recyclable pufs: Logically reconfigurable pufs. *J. Cryptogr. Eng.* **1**, 177 (2011).
44. Shah, N., Alam, M., Sahoo, D. P., Mukhopadhyay, D. & Basu, A. A 0.16 pJ/bit recurrent neural network based PUF for enhanced machine learning attack resistance. in *Proceedings of the 24th Asia and South Pacific Design Automation Conference* 627–632 (2019).
45. Uddin, M., Majumder, M. B. & Rose, G. S. Robustness analysis of a memristive crossbar PUF against modeling attacks. *IEEE Trans. Nanotechnol.* **16**, 396–405 (2017).
46. Rührmair, U. *et al.* Modeling attacks on physical unclonable functions. in *Proceedings of the 17th ACM conference on Computer and communications security* 237–249 (2010).
47. Delvaux, J. Machine-learning attacks on polypufs, ob-pufs, rpufs, lhs-pufs, and puf–fsms. *IEEE Trans. Inf. Forensics Secur.* **14**, 2043–2058 (2019).
48. Nili, H. *et al.* Hardware-intrinsic security primitives enabled by analogue state and nonlinear conductance variations in integrated memristors. *Nat. Electron.* **1**, 197–202 (2018).
49. Allen, P. E. & Holberg, D. R. *CMOS analog circuit design*. (Elsevier, 2011).
50. Lim, D. *et al.* Extracting secret keys from integrated circuits. *IEEE Trans. Very Large Scale Integr. Syst.* **13**, 1200–1205 (2005).
51. Cao, Y., Liu, C. Q. & Chang, C. H. A low power diode-clamped inverter-based strong physical unclonable function for robust and lightweight authentication. *IEEE Trans. Circuits Syst. I Regul. Pap.* **65**, 3864–3873 (2018).
52. Wang, Z. *et al.* Current mirror array: A novel circuit topology for combining physical unclonable function and machine learning. *IEEE Trans. Circuits Syst. I Regul. Pap.* **65**, 1314–1326 (2017).
53. Miller, A., Shifman, Y., Weizman, Y., Keren, O. & Shor, J. A Highly Reliable SRAM PUF with a Capacitive Preselection Mechanism and pre-ECC BER of 7.4 E-10. in *2019 IEEE Custom*

Integrated Circuits Conference (CICC) 1–4 (IEEE, 2019).

54. Pang, Y. *et al.* Optimization of RRAM-based physical unclonable function with a novel differential read-out method. *IEEE Electron Device Lett.* **38**, 168–171 (2017).

55. Liu, R., Wu, H., Pang, Y., Qian, H. & Yu, S. A highly reliable and tamper-resistant RRAM PUF: Design and experimental validation. in *2016 IEEE International Symposium on Hardware Oriented Security and Trust (HOST)* 13–18 (IEEE, 2016).

56. Pang, Y. *et al.* 25.2 A reconfigurable RRAM physically unclonable function utilizing post-process randomness source with< 6× 10− 6 native bit error rate. in *2019 IEEE International Solid-State Circuits Conference-(ISSCC)* 402–404 (IEEE, 2019).

57. Jiang, H. *et al.* A provable key destruction scheme based on memristive crossbar arrays. *Nat. Electron.* **1**, 548–554 (2018).
